# New Genotypes and Genomic Regions for Resistance to Wheat Blast in South Asian Germplasm

**DOI:** 10.3390/plants10122693

**Published:** 2021-12-08

**Authors:** Chandan Roy, Philomin Juliana, Muhammad R. Kabir, Krishna K. Roy, Navin C. Gahtyari, Felix Marza, Xinyao He, Gyanendra P. Singh, Aakash Chawade, Arun K. Joshi, Pawan K. Singh

**Affiliations:** 1 Department of Plant Breeding and Genetics, Bihar Agricultural University, Sabour 813210, India; chandan.roy43@gmail.com; 2 BISA/CIMMYT-India, NASC Complex, DPS Marg, New Delhi 110012, India; p.juliana@cgiar.org (P.J.); a.k.joshi@cgiar.org (A.K.J.); 3 Bangladesh Wheat and Maize Research Institute (BWMRI), Nashipur, Dinajpur 5200, Bangladesh; rezaulw@yahoo.com (M.R.K.); rkrishnaroy666@gmail.com (K.K.R.); 4 ICAR–Vivekanand Parvatiya Krishi Anushandhan Sansthan, Almora 263601, India; navin.gahtyari@icar.gov.in; 5 Instituto Nacional de Innovación Agropecuaria y Forestal (INIAF), La Paz, Bolivia; femarza@hotmail.com; 6 International Maize and Wheat Improvement Center (CIMMYT), Apdo. Postal 6-641, Mexico DF 06600, Mexico; x.he@cgiar.org; 7 ICAR—Indian Institute of Wheat and Barley Research, Karnal, Maharaja Agarsain Marg, P.O. Box 158, Karnal 132001, India; GP.Singh@icar.gov.in; 8 Department of Plant Breeding, Swedish University of Agricultural Sciences, 23053 Alnarp, Sweden; aakash.chawade@slu.se

**Keywords:** GWAS, SNPs, genotyping-by-sequencing, wheat blast

## Abstract

Wheat blast (WB) disease, since its first identification in Bangladesh in 2016, is now an established serious threat to wheat production in South Asia. There is a need for sound knowledge about resistance sources and associated genomic regions to assist breeding programs. Hence, a panel of genotypes from India and Bangladesh was evaluated for wheat blast resistance and a genome-wide association study (GWAS) was performed. Disease evaluation was done during five crop seasons—at precision phenotyping platform (PPPs) for wheat blast disease at Jashore (2018–19), Quirusillas (2018–19 and 2019–20) and Okinawa (2019 and 2020). Single nucleotide polymorphisms (SNP) across the genome were obtained using DArTseq genotyping-by-sequencing platform, and in total 5713 filtered markers were used. GWAS revealed 40 significant markers associated with WB resistance, of which 33 (82.5%) were in the 2NS/2AS chromosome segment and one each on seven chromosomes (3B, 3D, 4A, 5A, 5D, 6A and 6B). The 2NS markers contributed significantly in most of the environments, explaining an average of 33.4% of the phenotypic variation. Overall, 22.4% of the germplasm carried 2NS/2AS segment. So far, 2NS translocation is the only effective WB resistance source being used in the breeding programs of South Asia. Nevertheless, the identification of non-2NS/2AS genomic regions for WB resistance provides a hope to broaden and diversify resistance for this disease in years to come.

## 1. Introduction

Wheat blast (WB) caused by *Magnaporthe oryzae* pathotype *Triticum* (MoT) is a devastating disease found in warm and humid regions [1]. This pathogen is a highly variable ascomycetes fungus with several pathotypes known to infect almost all cereals including wheat [2]. Wheat infected by MoT was for the first time identified in the Parana state of Brazil in 1985, and the disease subsequently spread to its adjoining countries including Bolivia, Paraguay and Argentina [2,3,4]. Recently, WB has been reported in Bangladesh and Zambia [5,6], becoming a threat to wheat production in South Asia, Africa and beyond. A study revealed that MoT in Bangladesh belongs to South American lineage [7]. Nearly 6.9 mha of wheat growing areas in India, Pakistan and Bangladesh are vulnerable to WB, and an estimated loss of 886 thousand metric tons of wheat worth 132 million USD has been projected with a conservative estimate of 5% WB severity [8].

MoT infects all above-ground parts of wheat, but the most distinguishable symptom is complete or partially bleached spikes, and sometimes elliptical lesions appear on the leaf or culms [9]. Depending on the severity, WB can cause yield loss up to 100% [10,11,12] in susceptible cultivars and in South America yield loss of 40–100% is often reported [13]. Blast severity varied greatly depending on weather conditions, cultivar, and the plant organ which is infected [14,15]. Disease infection is mostly favored by temperatures of 25–30 °C, humidity > 80% and leaf wetness period > 25–40 h during the anthesis and grain filling stage [16,17].

Chemical control is expensive and detrimental to the environment and has low efficiency under high disease pressure [18]. Besides, resistance against some of the fungicides has been reported in MoT isolates from Brazil [19]. Therefore, the use of resistant varieties is considered the most effective strategy to manage this disease [20]. Despite extensive efforts in identifying WB resistance genes, only ten resistance genes have been identified to date that includes *Rmg1* through to *Rmg8, RmgTd(t)* and *RmgGR119* [1,20]. Among the resistance genes, *Rmg1, Rmg4* and *Rmg5* are not effective against MoT isolates, while the genes *Rmg2, Rmg3* and *Rmg7* are rendered ineffective by recent MoT isolates [21,22]. Only *Rmg8* and *RmgGR119* remain to be further tested for their effectiveness in commercial cultivars [23,24] across different environments. A translocated segment from the 2NS chromosome of *Aegilops ventricosa* has conferred resistance against MoT isolates [25]. This 2NS segment was first introduced to the winter wheat cultivar VPM1 in the process of transferring an eyespot resistance gene from *Ae. ventricose*, which eventually also carried a translocated segment at the telomeric end of chromosome 2AS [26]. The 2NS segment is reported to explain phenotypic variation up to 71.8% in CIMMYT germplasm [27], but the resistance is dependent on genetic background [28]. However, there are reports of the breakdown of 2NS WB resistance by the new MoT isolates [1]; hence identification of the alternative genes of resistance is of paramount importance for the development of durable WB resistant varieties.

WB resistance is quantitative in nature that involves varying degrees of resistance expression depending upon the genotype and genotype × environment interaction [29]. Studies reporting quantitative trait loci (QTLs) mapping for WB are limited. QTL mapping in the Caninde#1/Alondra biparental population identified 2NS as the main source of resistance, explaining phenotypic variation ranging from 22.4 to 50.1%, along with minor QTLs on chromosome 2BL, 3AL, 4BS, 4DL and 7BS [30]. GWAS using 1106 CIMMYT breeding lines identified 36 marker-trait associations (MTAs) on chromosomes 2AS, 3BL, 4AL and 7BS [27]. Another study using 384 spring wheat lines from different countries identified significant WB MTAs on chromosomes 2AS, 1A and 1D [31]. Characterization of South Asian wheat germplasms for WB resistance is largely unexplored. Recently, GWAS using genotypes from CIMMYT, India, Bangladesh and Nepal identified significant MTAs on chromosomes 2AS (2NS/2AS translocation region), 1BS, 6BS and 7BS [29].

With the growing importance of WB in South Asia, the Indian Council of Agricultural Research (ICAR) has started a flagship program with CIMMYT, Mexico in the year 2017 to screen Indian wheat germplasm for WB under precision phenotyping platforms (PPP) in Bolivia and Bangladesh. Since then, each year, a set of Indian germplasm has been routinely screened for WB resistance at the PPPs. In the present study, a set of wheat germplasms from India and Bangladesh were characterized at the PPPs to identify the resistant genotypes and map WB-associated genomic regions to develop and deploy resistant genotypes to restrict WB spread.

## 2. Results

### 2.1. Phenotypic Variation and Heritability of WB and Its Association with Other Traits

Analysis of variance revealed significant genetic variation (*p* < 0.0001) for WB among the tested genotypes in all the locations (Table S1). Effect of years in Okinawa (*p* < 0.0001) and Quirusillas (*p* < 0.0001); sowing within year (*p* < 0.0001) and genotype × year interaction (*p* < 0.0001) in Okinawa were highly significant. Heritability was highest at Okinawa (0.79), while lowest at Jashore (0.41). Population distribution for the WB index was typically bimodal except for Jash19a, Oki20a and Quir20a where disease pressure was relatively lower (Figure S1).

Mean WB index ranged from 30.3 ± 2.3 (Quir20a) to 63.3 ± 2.6 (Oki19a), with a grand mean index across experiments being 46.9 ± 1.8 (Table S2). In Quirusillas and Jashore, the disease pressure was higher in the second sowing, but in Okinawa, it did not differ much between sowings (Table S3). Urubo and Bari Gom 33 were resistant to WB, while checks, Altax and Bari Gom 26 were highly susceptible. Overall, 24.7% of the lines were resistant (mean WB index < 10%), and the proportion of resistant lines in the Indian population (38.3%) was higher than in Bangladesh (9.7%) wheat germplasm (Table S4).WB was often positively associated with days to heading (DH) (r = 0.46 in Quir19a), while its association with plant height (PH) was mostly non-significant (Table S5). We observed a significant association of WB index across experiments, with only a few exceptions (Table S6). Within the same location, the correlation between two sowing dates and between two years of the evaluation was also significant (*p* < 0.001).

### 2.2. Population Structure and GWAS Analysis

**Figure 1:**
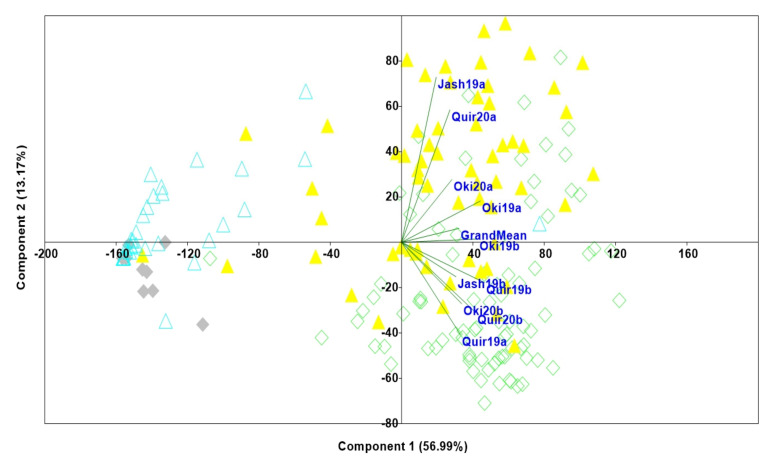
Principle component analysis of 187 wheat lines for blast index in all the environments. The genotypes of Indian and Bangladesh origin are depicted in triangle and diamond shapes, respectively. Genotypes with blue colored unfilled triangle and grey colored filled diamond are 2NS positive from India and Bangladesh, respectively.

Principal component analysis using the WB index revealed most of the genotypes from Bangladesh were grouped together while those from India were more scattered. The percentage of phenotypic variation explained (PVE) by the PC1 and PC2 was 56.99% and 13.17% respectively (Figure 1). Genotypes with and without the 2NS segment were clearly separated in the dimension of PC2. Among the 2NS positive genotypes, 38% carried Milan or its derivative line Kachu in their pedigree, indicating a similar genetic background for WB resistance.

GWAS was carried out for individual environments and the combined dataset. Altogether, 40 significant markers (*p* < 0.001) trait association (MTAs) were detected (Table S7). Among them 33 MTAs were from the 2A chromosome, and one MTA each on the other seven chromosomes (3B, 3D, 4A, 5A, 5D, 6A and 6B) (Table 1).

**Figure 2:**
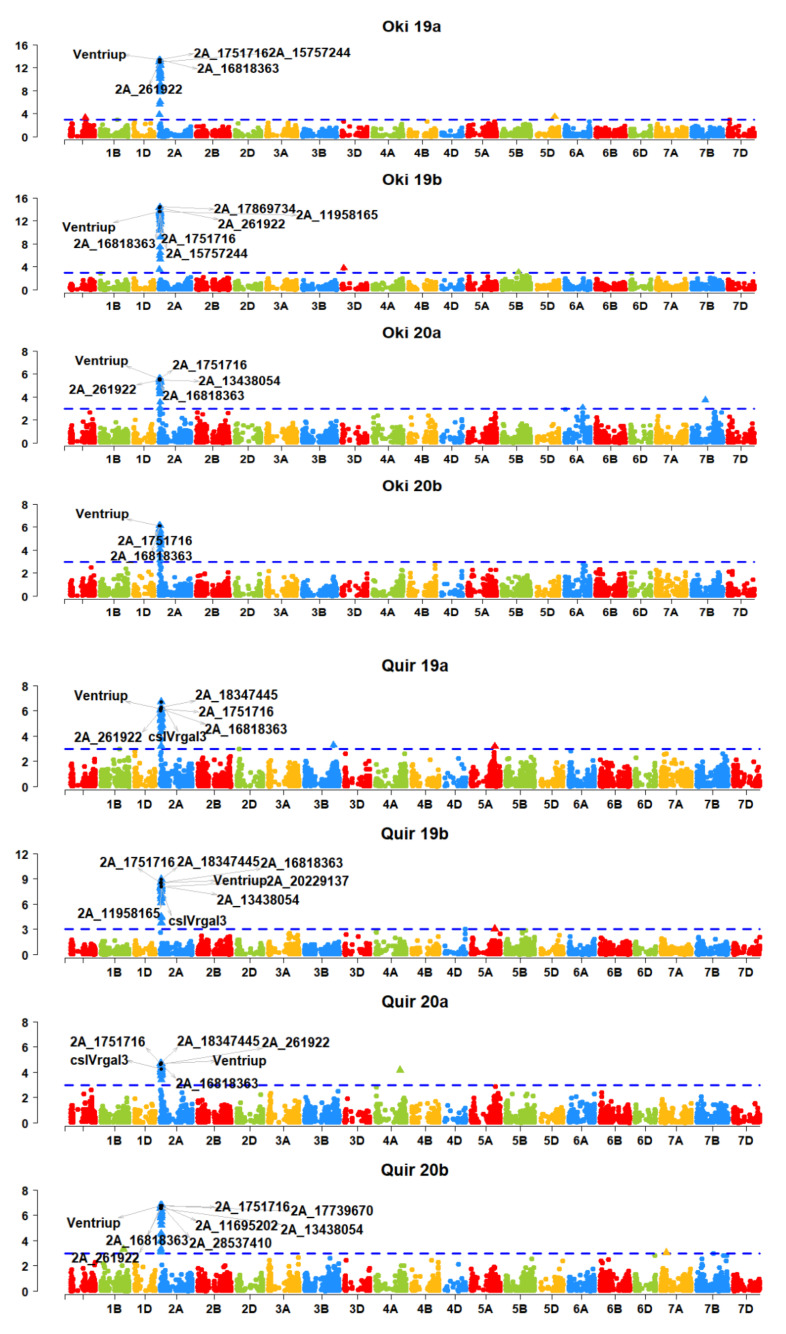

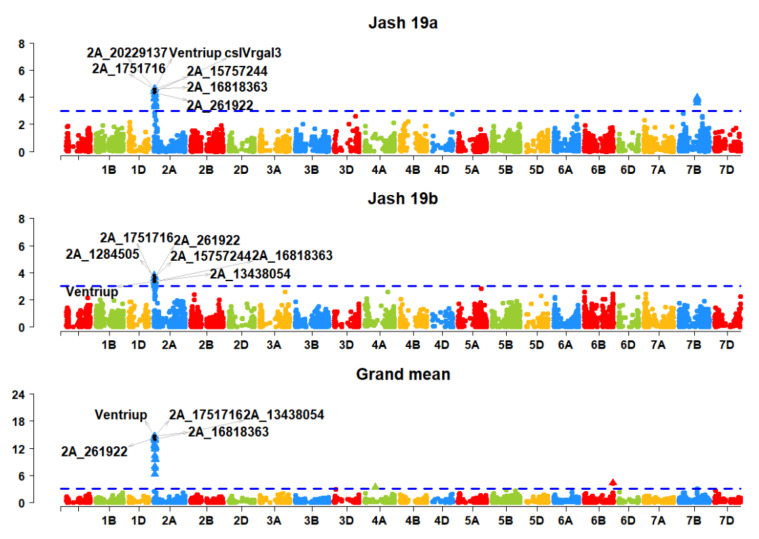
Manhattan plots showing markers significantly associated with WB in all the environments and grand mean. Key markers in each environment are visualized. A Bonferroni α level of 0.20 was used to determine the significant markers.

All the significant SNPs on 2AS including the four STS markers specific to the 2NS/2AS translocation were significant in more than one environment (Figure 2); 2A_16818363 in Oki19a, Oki20a Oki20b and 2A_17869734 in Oki19b; cslVrgal3 in Quir19a; 2A_18347445 in Quir19b; 2A_18347445 in Quir20a; 2A_ 11695202, 2A_16818363 and 2A _28537410 in Quir20b, and 2A_1284505 in Jash19b were the most significant markers. Considering all the experiments and the combined data set, 2A_1751716 and Ventriup were among the highly significant markers obtained. No significant MTA was detected in Jash19a. MTAs other than those on chromosome 2A were expressed only in one environment or in the grand mean. Two MTAs on 4A (4A_263484695) and 6B (6B_721501177) were significant only in the grand mean.

It is noteworthy that none of the markers other than on the 2A chromosome were significant in Jash19b, Oki20b and Quir20b. Marker additive effects on blast index ranged from 1.2 for 5A_582828314 and 5D_451911605 to 22.2 for 2A_18665467. The higher additive effect ranging from 4.4–22.2 was recorded for the MTAs on the 2AS chromosome with the average additive effect of 19.0 ± 4.3. Marker R^2^ was higher for the MTAs on 2A, ranging from 0.04 for 2A_2797789 to 0.4 for 2A_19743082 with average R^2^ value of 0.33 ± 0.08. Among the non-2A markers, 6B_721501177 had the highest R^2^ value of 0.09 (Table S7).

Linkage disequilibrium analysis revealed that markers on the same chromosome, particularly 2A showed higher R^2^ values (Figure S2). One of the markers, 3D_38060848 showed high LD with the markers on the 2A chromosome; however, no result was obtained related to 2A when the marker sequence was BLAST with the reference genome sequence (IWGSC RefSeq v1.0).

### 2.3. Effect of the 2NS/2AS Translocation on WB

**Figure 3:**
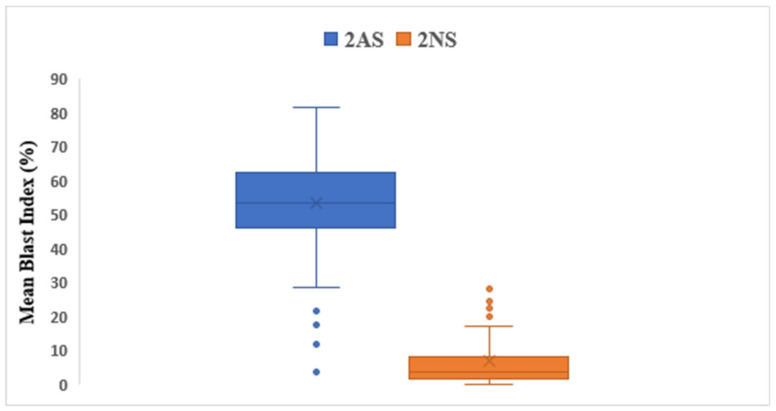
Effects of the 2AS and 2NS segments on grand mean blast index.

Among the significant resistant loci, the 2NS/2AS chromosomal region was consistently significant across the environments. Overall, 22.4% of the panel carried the 2NS segment, including 35.1% of the Indian population and 9.6% of Bangladesh. The presence of 2NS in the genotypes significantly reduced the WB index (Figure 3). The average WB index in the 2NS positive lines was 6.8% while in the non-2NS lines, it was 58.3%. Markers located on the 2NS/2AS translocation region were significant in most of the tested environments.

## 3. Discussion

In the present study, 187 wheat genotypes were screened for WB in Bolivia and Bangladesh. Significant association of WB score in Bangladesh (Jashore), with those from Bolivia (Quirusillas and Okinawa) indicates the effectiveness of WB screening at Jashore. However, the lack of a strong correlation between Bolivia and Bangladesh may be attributed to the influence of climatic factors and the difference in virulence of MoT isolates on WB as revealed from a significant genotype × environment interaction. Significant genetic variation among the tested lines and moderate heritability indicated the possibility of selection of resistant genotypes.

GWAS revealed 40 significant MTAs associated with WB; but apart from those on 2NS/2AS, none was significant in more than one environment, indicating that 2NS is the primary stable source of WB resistance in the germplasm used. The 2NS segment is characterized as rich in genes with nucleotide binding leucine rich repeats (NLR) that confers resistance against multiple diseases along with nematode resistance [32], lodging resistance [33] and yield advantage [34]. Comparing the position of the significant markers through BLAST to the IWGSC RefSeq v1.0 genome sequence of Chinese Spring revealed a range from 0.26Mb to 29.12Mb delimited by 2A_261922 and 2A_29121496, respectively, mostly falling into the 2NS segment. An average phenotypic variation (PV) explained by the 2NS markers across the environments was 33.4%, which is similar to those reported in previous studies, e.g., 37.2% [27] and 26 to 50% [29]. Most of the lines carrying the 2NS segment were either derived from Milan or its derivative Kachu. Milan has shown a high level of resistance against WB and is extensively used for cultivars development [35]. Several Milan-based cultivars like Sausal CIAT, CD 116 and Caninde 1 were released in South America and are popular among the farmers [25,35]. It is noteworthy that eight lines in the Indian and one in the Bangladesh population were 2NS positive but scored higher (>10) WB index. Among the 2NS positive lines, K1315 from India scored the highest WB index of 33.7% (Table S8), implying that the resistance of 2NS could be dependent on the genetic background, in accordance with previous observations [25]. The possibility of recombination occurring in these lines could result in a false positive for the presence of 2NS translocation.

Identification of the gene(s) or QTLs other than 2NS is of utmost importance to diversify resistance sources. Presently, 2NS is the only reliable source of resistance against WB, which increases the vulnerability for the disease outbreak if there is a breakdown of this resistance. Already, the 2NS-virulent isolates are emerging rapidly in South America [1,2] and may spread further. Besides, 2NS-based genotypes have shown large phenotypic variation and often confer insufficient resistance under high disease pressure [25,30]. Therefore, pyramiding 2NS with QTLs of minor effects would increase the resistance and durability [29,30]. This appears feasible since non-2NS lines with moderate WB resistance (DBW 39 and BAW-1272) were identified in this study. These two lines do not possess a 2NS segment but exhibited a mean WB index of 11.3 and 13.3%, respectively. Similarly, other researchers have reported resistance to moderately resistant genotypes without 2NS translocation under field and greenhouse conditions [21,29,36]. Although, the performance of these genotypes was not consistent over the environments, varying from highly resistant to moderately susceptible, in accordance with previous studies [1,37]. Nevertheless, these genotypes can be used as alternative WB resistant sources in wheat breeding to broaden and diversify the genetic basis of WB resistance.

With the first report of WB in Bangladesh, one of the most important questions was whether the disease could spread to its neighboring countries or not. After the initial occurrence in eight districts of Bangladesh, the disease spread further to its adjoining districts in the subsequent years even under unfavorable conditions [1,38,39]. This raised a serious concern for the vulnerable areas in India and other countries in the vicinity. The Indian Council of Agricultural Research (ICAR) adopted several preventive measures, including wheat holiday and strict quarantine, to avoid the introduction of contaminated seeds and also encouraged fast track release and promotion of WB resistant cultivars for commercial cultivation in vulnerable areas [40]. Within a short period of five years, fourteen WB resistant genotypes were released for commercial cultivation in India, among which WB 02, HD 3171, DBW 173, DBW 187 also contain high grain iron and zinc concentration and DBW 88 has high grain protein content (Table 2).

DBW 187 has become a pan Indian wheat variety due to its release in the three major zones of India that grow around 25 m ha of wheat. Bangladesh has released blast resistant varieties BARI Gom 33 and WMRI Gom 3 for commercial cultivation [1,36,41]. In India, so far there is no report of MoT occurring on wheat or any other host. *Magnaporthe oryzae* pathotype *Oryzae* (MoO) infecting rice is prevalent in India, and although MoO is generally regarded as avirulent to wheat under field conditions, a recent report from China showed its infection in wheat under laboratory conditions [42]. Therefore, continuous monitoring of WB-like symptoms in wheat fields, especially those in the regions of India and Nepal that border with Bangladesh, is important to prevent the WB epidemic in South Asia. In the present study, 2NS is the only resistance source observed in the assayed genotypes from India and Bangladesh, imparting an urgent need for further studies to identify new genes and to diversify resistant sources.

## 4. Materials and Methods

### 4.1. Plant Materials

A panel of 187 genotypes comprising 94 from India and 93 from Bangladesh were used in this study. The majority of these genotypes are the most recent advanced lines in the ongoing breeding programs in the two countries.

### 4.2. Experimental Design and Field Phenotyping

The experiments were conducted at three locations, including Quirusillas and Okinawa in the Department of Santa Cruz, Bolivia and Jashore in the Khulna division of Bangladesh. In Quirusillas and Okinawa, wheat seasons are warm and humid with frequent rainfall, while Jashore is warm and dry with occasional rainfall. At each location, wheat was sown twice at an interval of around 14 days. At Quirusillas and Jashore, sowing was done in December, while at Okinawa it was done in May. Overall ten different experiments were planted, two at Bangladesh (Jashore) and eight at Bolivia (four each at Quirsusillas and Okinawa). These ten experiments were set up as Jashore (year 2018–19; denoted as Jas19a for the first sowing and Jash19b for the second); Quirusillas—2018–19 (Quir19a and Quir19b), and 2019–20 (Quir20a and Quir20b); Okinawa—2019 (Oki19a and Oki19b) and 2020 (Oki20a and Oki20b). In each environment wheat was sown in double row plots of 1-m length with 20-cm spacing. The wheat varieties BARI Gom 33 and BARI Gom 26 at Jashore; Urubo and Atlax at Quirusillas and Okinawa were sown as resistant and susceptible checks, respectively. Resistance sources of BARI Gom 33 and Urubo were Kachu and Milan, respectively. Wheat was inoculated twice, at anthesis and two days after anthesis, at each location with highly aggressive MoT isolates using a backpack sprayer. Inoculum suspension was prepared using an isolate mixture of QUI1505, QUI1601, QUI1612, OKI1503, and OKI1704 for spraying at Quirusillas and Okinawa, and isolates BHO17001, MEH17003, GOP17001.2, RAJ17001, CHU16001.3, and JES16001 at Jashore, at a concentration of approximately 80,000 conidia mL^−1^ of water [30]. An automatic misting system was set up for spraying water for 15 min every hour from 9 am to 5 pm at Jashore and from 8 am to 5 pm at Quirusillas and Okinawa after the first inoculation throughout the period (4–5 weeks) of disease establishment, to maintain high humidity favoring WB development.

Evaluation for WB was carried out 18–21 days after the first inoculation, for which ten spikes were tagged randomly in each plot. WB incidence was recorded as a proportion of infected spikes and severity as the proportion of infected spikelets. Blast index was calculated as incidence × severity for each genotype and mean of WB index across all the environment was also calculated. In addition, plant height and days to heading were also recorded. Statistical analysis was carried out using the statistical software PROC GLM Module of SAS ver 9.2. Heritability was estimated using the formula H^2^ = σ^2^_g_/(σ^2^_g_ + σ^2^_e_/s) for Jashore and H^2^ = σ^2^_g_/(σ^2^_g_ + σ^2^_g*y_/y + σ^2^_g*s_/s + σ^2^_e_/sy) for Quirusillas and Okinawa in which σ^2^_g_ stands for genetic variance, σ^2^_g*y_ for genotype-by-year interaction, σ^2^_e_ for error variance, y for the number of years, and s for the number of plantings. Population structure was assessed through principal component analysis (PCA) using blast index, calculated in the software PAST version 3.01 [43].

### 4.3. Genotyping

Genomic DNA was isolated from leaves of two weeks old seedlings using the CTAB method. Single nucleotide polymorphism (SNP) genotyping of the panel was carried out using DArTSeq genotyping-by-sequencing platform [44]. Markers were filtered with minor allele frequency (MAF) of more than 10%, missing data less than 60% and heterozygosity less than 10% for further analysis. Initially, 58,037 SNP markers were generated, and after filtering 5713 markers were used for GWAS. Four STS markers *Ventriup-LN2* [45], *cslVrgal3* [30], *WGGB156* and *WGGB159* [46] were used to confirm the presence of 2NS segment in the genotypes. The linkage disequilibrium k-nearest neighbor genotype imputation method was used for the imputation of genotypic data [47].

### 4.4. GWAS Analysis

Marker trait associations were obtained in the software TASSEL version 5 [48] using the mixed linear model with the optimum level of compression and default parameters. Population structure was calculated using PCA, where the first two principal components were used as fixed effects. In addition, kinship relationships among the individuals were computed using the centered identity-by-state method which was used as a random effect in the mixed linear model. The *p* values, additive effects and marker R^2^ values were estimated and Manhattan plots with –log_10_p-values were then plotted using the R package CMplot [49]. Significant markers were identified using the Bonferroni method for multiple testing corrections at an α level of 0.20.

## 5. Conclusions

WB is emerging as a potential threat to wheat production in South Asia. The present study revealed 2NS is the only effective resistance source in the South Asian wheat germplasm evaluated in the current study. Deployment of 2NS-carrying resistance lines can be effective in managing the WB disease. Identification and utilization of new sources of resistance will further diversify the resistance gene pool and prevent the disease spread.

## Figures and Tables

**Table 1:** Significant SNPs associated with WB resistance.

SNP	Chromosome	Physical Position	*p*-Value	Additive Effect	PVE (%)
Multiple SNPs	2A	261922–29121496	2.37 × 10^−15^–8.30 × 10^−10^	(−)22.23–22.13	4.87–40.19
1003077	3B	711240574	5.45 × 10^−4^	−2.87	1.443
990037	3D	38060848	1.80 × 10^−4^	−7.02	7.387
2259170	4A	263484695	4.30 × 10^−4^	1.48938	8.622
1714015	5A	582828314	6.82 × 10^−4^	−3.02	2.976
5324898	5D	451911605	3.66 × 10^−4^	−3.70	1.745
1213518	6A	433695606	8.61 × 10^−4^	−2.4756	1.098
3024082	6B	721501177	5.31 × 10^−5^	−2.57015	9.13

**Table 2:** List of 2NS positive varieties of wheat from India recommended for commercial cultivation.

S.N.	Specific Name	Mean WB Index	Remarks
1.	DBW 252	2.2	Resistance to foliar diseases and tolerance to drought, potential yield of 55.6 q/ha
2.	HD 3171	2.8	Tolerance to drought, potential yield of 46.33 q/ha, higher iron content (47.1 ppm ^1^)
3.	DBW 88	2.0	Higher protein content (13.8%) and perfect 10/10 Glu-1 score with with yield potential up to 69.9 q/ha
4.	DBW 168	9.3	Potential yield 70.1 q/ha with very good for chapati
5.	DBW 173	0.0	Heat tolerant suitable for late sown with potential yield 57.0 q/ha, iron 40.7 ppm
6.	DBW 187	0.2	Potential yield 95.9 q/ha with higher iron content (43.1 ppm)
7.	DBW 222	8.1	Potential yield 82.1 q/ha
8.	HD 2967	3.2	Average yield 44 q/ha
9.	HD 3043	0.6	Potential yield 66.0 q/ha
10.	HD 3249	9.8	Potential yield 67.5 q/ha; higher grain zinc (37 ppm) and iron content (42.5 ppm)
11.	WH 1105	3.0	Potential yield 71.6 q/ha
12.	PBW 677	0.7	Average yield 57.7 q/ha
13.	PBW 752	24.6	Potential yield 65.4q/ha, protein (12.4%)
14.	WB 2	5.3	Potential yield-58.9q/ha, rich in zinc (42.0 ppm) and iron (40.0 ppm)

^1^ parts per million.

## Data Availability

The original contributions presented in the study are publicly available. This data can be found here: https://hdl.handle.net/11529/10548634 (accessed on 3 December 2021).

## Supplementary Materials

The following are available online at https://www.mdpi.com/article/10.3390/plants10122693/s1, Table S1: Analysis of variance and heritability estimates of WB, Table S2: Statistical parameters for WB in each environments and grand mean, Table S3: WB index of genotypes in different environments and grand mean, Table S4: Proportion of resistant genotypes in different environments (WB score 0 at individual environment and <10 in grand mean), Table S5: Association of WB index with plant height (PH) and days to heading (DH) in different environments, Table S6: Association among the environments for WB, Table S7: Chromosome, position, *p*-value, additive effect, and R2 value for significant markers for WB resistant over the environments and grand mean, Table S8: List of 2NS positive genotypes from India and Bangladesh, Figure S1: Histogram of WB index obtained in different environments, Figure S2: Linkage disequilibrium (LD) plot showing relationship among the significant markers with 2A based on their R^2^ and *p*-value.

## References

[1] Singh P.K., Gahtyari N.C., Roy C., Roy K.K., He X., Tembo B., Xu K., Juliana P., Sonder K., Kabir M.R. (2021). Wheat blast: A disease spreading by intercontinental jumps and its management strategies. Front. Plant Sci..

[2] Ceresini P.C., Castroagudín V.L., Rodrigues F.Á., Rios J.A., Eduardo Aucique-Pérez C., Moreira S.I., Alves E., Croll D., Maciel J.L.N. (2018). Wheat blast: Past, present, and future. Annu. Rev. Phytopathol..

[3] Barea G., Toledo J. (1996). Identificación y zonificación de Pyricularia o brusone (*Pyricularia oryzae*) en el cutivo de trigo en el departamento de Santa Cruz. Centro de Investigación Agrícola Tropical. Informe Tecnico. Proyecto de Investigacion Trigo.

[4] Perelló A., Martinez I., Molina M. (2015). First report of virulence and effects of *Magnaporthe oryzae* isolates causing wheat blast in Argentina. Plant Dis..

[5] Malaker P.K., Barma N.C.D., Tiwari T.P., Collis W.J., Duveiller E., Singh P.K., Joshi A.K., Singh R.P., Braun H.-J., Peterson G.L. (2016). First report of wheat blast caused by *Magnaporthe oryzae* pathotype *triticum* in Bangladesh. Plant Dis..

[6] Tembo B., Mulenga R.M., Sichilima S., M’siska K.K., Mwale M., Chikoti P.C., Singh P.K., He X., Pedley K.F., Peterson G.L. (2020). Detection and characterization of fungus (*Magnaporthe oryzae* pathotype *Triticum*) causing wheat blast disease on rain-fed grown wheat (*Triticum aestivum* L.) in Zambia. PLoS ONE.

[7] Islam M.T., Croll D., Gladieux P., Soanes D.M., Persoons A., Bhattacharjee P., Hossain M.S., Gupta D.R., Rahman M.M., Mahboob M.G. (2016). Emergence of wheat blast in Bangladesh was caused by a South American lineage of *Magnaporthe oryzae*. BMC Biol..

[8] Mottaleb K.A., Singh P.K., Sonder K., Kruseman G., Tiwari T.P., Barma N.C.D., Malaker P.K., Braun H.J., Erenstein O. (2018). Threat of wheat blast to South Asia’s food security: An *ex-ante* analysis. PLoS ONE.

[9] Igarashi S., Saunders D.A. (1990). Update on wheat blast (*Pyricularia oryzae*) in Brazil. Wheat for the Nontraditional Warm Areas. A Proceedings of the International Conference, Foz do Iguaçu, Brazil, 29 July–3 August 1990.

[10] Goulart A.C.P., Paiva F.A. (1992). Incidence of (*Pyricularia oryzae*) in different wheat cultivars under field conditions. Fitopatol. Bras..

[11] Goulart A.C.P., Paiva F.A. (2000). Perdas no rendimento de graos de trigo causadas por *Pyricularia grisea* nos anos de 1991 e 1992, no Mato Grosso do Sul. Summa Phytopathol..

[12] Duveiller E., He X., Singh P.K., Bonjean A.P., Angus W.J., van Ginkel M. (2016). Wheat blast: An emerging disease in South America potentially threatening wheat production. The World Wheat Book Vol. 3.

[13] McDonald B.A., Stukenbrock E.H. (2016). Rapid emergence of pathogens in agro-ecosystems: Global threats to agricultural sustainability and food security. Philos. Trans. R. Soc. B Biol. Sci..

[14] Goulart A.C.P., Sousa P.G., Urashima A.S. (2007). Damages in wheat caused by infection of *Pyricularia grisea*. Summa Phytopathol..

[15] Urashima A.S., Grosso C., Stabili A., Freitas E., Silva D., Netto D., Franco I., Bottan M., Wang G.L., Valent B. (2009). Effect of *Magnaporthe grisea* on seed germination, yield and quality of wheat. Advances in Genetic, Genomics and Control of Rice Blast Disease.

[16] Cardoso C.A.D.A., Reis E.M., Moreira E.N. (2008). Development of a warning system for wheat blast caused by *Pyricularia grisea*. Summa Phytopathol..

[17] Singh D.P. (2017). Wheat blast—A new challenge to wheat production in South Asia. Indian Phytopathol..

[18] Urashima A.S., Alves A.F., Silva F.N., Oliveira D., Gazaffi R. (2017). Host range, mating type and population structure of *Magnaporthe sp.* of a single barley field in São Paulo state, Brazil. J. Phytopathol..

[19] Castroagudín V.L., Ceresini P.C., De Oliveira S.C., Reges J.T.A., Maciel J.L.N., Bonato A.L.V., Dorigan A.F., McDonald B.A. (2015). Resistance to QoI fungicides is widespread in Brazilian populations of the wheat blast pathogen *Magnaporthe oryzae*. Phytopathology.

[20] Chowdhury A.K., Saharan M.S., Aggrawal R., Malaker P.K., Barma N.C.D., Tiwari T.P., Duveiller E., Singh P.K., Srivastava A.K., Sonder K. (2017). Occurrence of wheat blast in Bangladesh and its implications for South Asian wheat production. Indian J. Genet. Plant Breed..

[21] Cruppe G., Cruz C.D., Peterson G., Pedley K., Asif M., Fritz A., Calderon L., da Silva C.L., Todd T., Kuhnem P. (2020). Novel sources of wheat head blast resistance in modern breeding lines and wheat wild relatives. Plant Dis..

[22] Cruz C.D., Valent B. (2017). Wheat blast disease: Danger on the move. Trop. Plant Pathol..

[23] Anh V.L., Anh N.T., Tagle A.G., Vy T.T.P., Inoue Y., Takumi S., Chuma I., Tosa Y. (2015). *Rmg8*, a new gene for resistance to *Triticum* isolates of *Pyricularia oryzae* in hexaploid wheat. Phytopathology.

[24] Wang S., Asuke S., Vy T.T.P., Inoue Y., Chuma I., Win J., Kato K., Tosa Y. (2018). A new resistance gene in combination with *Rmg8* confers strong resistance against *triticum* isolates of *Pyricularia oryzae* in a common wheat landrace. Phytopathology.

[25] Cruz C.D., Peterson G.L., Bockus W.W., Kankanala P., Dubcovsky J., Jordan K.W., Akhunov E., Chumley F., Baldelomar F.D., Valent B. (2016). The 2NS translocation from *Aegilops ventricosa* confers resistance to the *Triticum* pathotype of *Magnaporthe oryzae*. Crop Sci..

[26] Badaeva E.D., Dedkova O.S., Koenig J., Bernard S., Bernard M. (2008). Analysis of introgression of *Aegilops ventricosa* Tausch. genetic material in a common wheat background using C-banding. Theor. Appl. Genet..

[27] Juliana P., He X., Kabir M.R., Roy K.K., Anwar M.B., Marza F., Poland J., Shrestha S., Singh R.P., Singh P.K. (2020). Genome-wide association mapping for wheat blast resistance in CIMMYT’s international screening nurseries evaluated in Bolivia and Bangladesh. Sci. Rep..

[28] Cardozo Téllez L., Chavez A., Bobadilla N., Pérez-Estigarribia P., Kohli M. (2018). Variable resistance of bread wheat (*Triticum aestivum*) lines carrying 2NS/2AS translocation to wheat blast. Plant Breed..

[29] He X., Juliana P., Kabir M.R., Roy K.K., Islam R., Marza F., Peterson G., Singh G.P., Chawade A., Joshi A.K. (2021). Screening and mapping for head blast resistance in a panel of CIMMYT and South Asian bread wheat germplasm. Front. Genet..

[30] He X., Kabir M.R., Roy K.K., Anwar M.B., Xu K., Marza F., Odilbekov F., Chawade A., Duveiller E., Huttner E. (2020). QTL mapping for field resistance to wheat blast in the Caninde#1/Alondra population. Theor. Appl. Genet..

[31] Cruppe G., Silva P., da Silva C.L., Peterson G., Pedley K.F., Cruz C.D., Asif M., Lollato R.P., Fritz A.K., Valent B. (2021). Genome-wide association reveals limited benefits of pyramiding the 1B and 1D loci with the 2NS translocation for wheat blast control. Crop Sci..

[32] Williamson V.M., Thomas V., Ferris H., Dubcovsky J. (2013). An *Aegilops ventricosa* translocation confers resistance against root-knot nematodes to common wheat. Crop Sci..

[33] Singh D., Wang X., Kumar U., Gao L., Noor M., Imtiaz M., Singh R.P., Poland J. (2019). High-throughput phenotyping enabled genetic dissection of crop lodging in wheat. Front. Plant Sci..

[34] Gao L., Koo D.-H., Juliana P., Rife T., Singh D., Lemes da Silva C., Lux T., Dorn K.M., Clinesmith M., Silva P. (2020). The *Aegilops ventricosa* 2NvS segment in bread wheat: Cytology, genomics and breeding. Theor. Appl. Genet..

[35] Kohli M.M., Mehta Y.R., Guzman E., de Viedma L., Cubilla L.E. (2011). *Pyricularia* blast-a threat to wheat cultivation. Czech J. Genet. Plant Breed..

[36] Roy K.K., Reza M.M.A., Muzahid-E-Rahman M., Anwar M.B., Kabir M.R., Malaker P.K., Barma N.C.D., Hossain M.I., He X., Chawade A. (2021). Evaluation of elite bread wheat lines for resistance to blast disease in Bangladesh. Euphytica.

[37] Wu L., He X., Kabir M.R., Roy K.K., Anwar M.B., Marza F., He Y., Jiang P., Zhang X., Singh P.K. (2021). Genetic sources and loci for wheat head blast resistance identified by genome-wide association analysis. Crop J..

[38] Islam M.T., Gupta D.R., Hossain A., Roy K.K., He X., Kabir M.R., Singh P.K., Khan M.A.R., Rahman M., Wang G.-L. (2020). Wheat blast: A new threat to food security. Phytopathol. Res..

[39] Mustarin K.E., Roy K.K., Rahman M.M.E., Reza M.M.A., Hossain M.I. (2021). Surveillance and monitoring of some major diseases of wheat in Bangladesh with special emphasis on wheat blast- a new disease in Bangladesh. J. Plant Pathol..

[40] Bishnoi S.K., Kumar S., Singh G.P. (2021). Wheat blast readiness of the Indian wheat sector. Curr. Sci..

[41] Hossain A., Mottaleb K.A., Farhad M., Deb Barma N.C. (2019). Mitigating the twin problems of malnutrition and wheat blast by one wheat variety, “BARI Gom 33”, in Bangladesh. Acta Agrobot..

[42] Shizhen W., Jiaoyu W., Zhen Z., Zhongna H., Xueming Z., Rongyao C., Haiping Q., Yanli W., Fucheng L., Guochang S. (2021). The risk of wheat blast in rice–wheat co-planting regions in China: MoO strains of *Pyricularia oryzae* cause typical symptom and host reaction on both wheat leaves and spikes. Phytopathology.

[43] Hammer Ø., Harper D.A.T., Ryan P.D. (2001). Past: Paleontological Statistics Software Package for Education and Data Analysis. Palaeontol. Electron..

[44] Li H., Vikram P., Singh R.P., Kilian A., Carling J., Song J., Burgueno-Ferreira J.A., Bhavani S., Huerta-Espino J., Payne T. (2015). A high density GBS map of bread wheat and its application for dissecting complex disease resistance traits. BMC Genom..

[45] Helguera M., Khan I.A., Kolmer J., Lijavetzky D., Zhong-qi L., Dubcovsky J. (2003). PCR assays for the Lr37-Yr17-Sr38 cluster of rust resistance genes and their use to develop isogenic hard red spring wheat lines. Crop Sci..

[46] Wang Y., Zhang H., Xie J., Guo B., Chen Y., Zhang H., Lu P., Wu Q., Li M., Zhang D. (2018). Mapping stripe rust resistance genes by BSR-Seq: *YrMM58* and *YrHY1* on chromosome 2AS in Chinese wheat lines Mengmai 58 and Huaiyang 1 are *Yr17*. Crop J..

[47] Money D., Gardner K., Migicovsky Z., Schwaninger H., Zhong G.-Y., Myles S. (2015). LinkImpute: Fast and accurate genotype imputation for nonmodel organisms. G3 Genes Genomes Genet..

[48] Bradbury P.J., Zhang Z., Kroon D.E., Casstevens T.M., Ramdoss Y., Buckler E.S. (2007). TASSEL: Software for association mapping of complex traits in diverse samples. Bioinformatics.

[49] Yin L., Zhang H., Tang Z., Xu J., Yin D., Zhang Z., Yuan X., Zhu M., Zhao S., Li X. (2021). rMVP: A Memory-efficient, Visualization-enhanced, and parallel-accelerated tool for genome-wide association study. Genom. Proteom. Bioinform..

