# Supplementary figures and images for: New Genotypes and Genomic Regions for Resistance to Wheat Blast in South Asian Germplasm
Source: Plants (Basel). 2021 Dec 8;10(12):2693. doi: 10.3390/plants10122693 (PMC8708018; doi:10.3390/plants10122693)

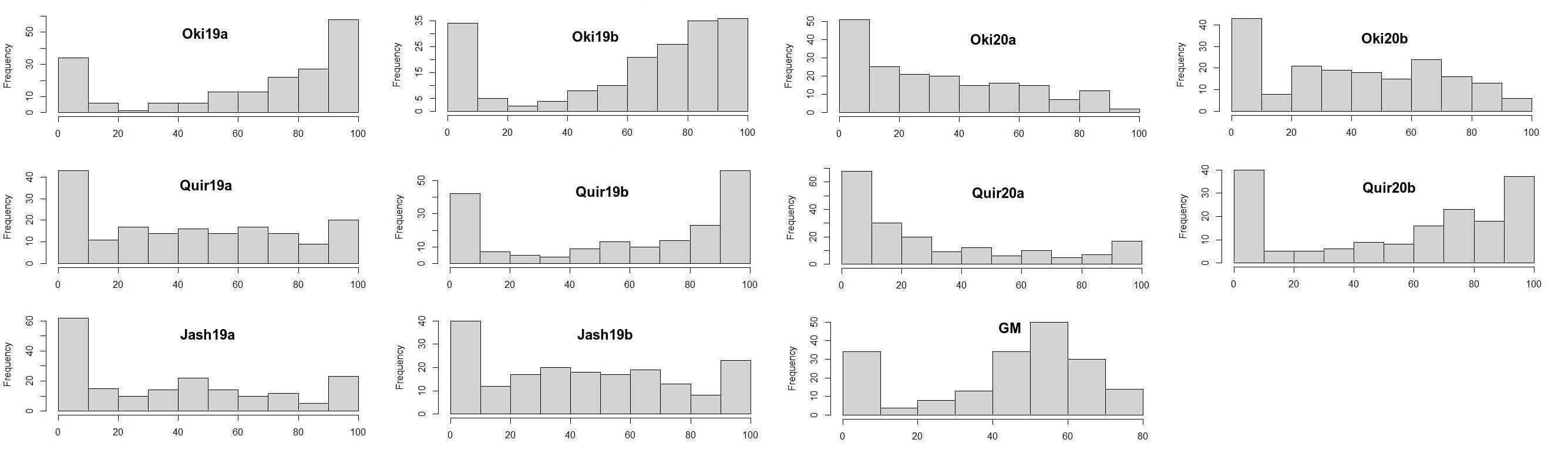

Supplement: Supplementary file 1 [file plants-10-02693-s001.zip › plants-1455551-supplementary/Supplementary Figure S1.jpg]

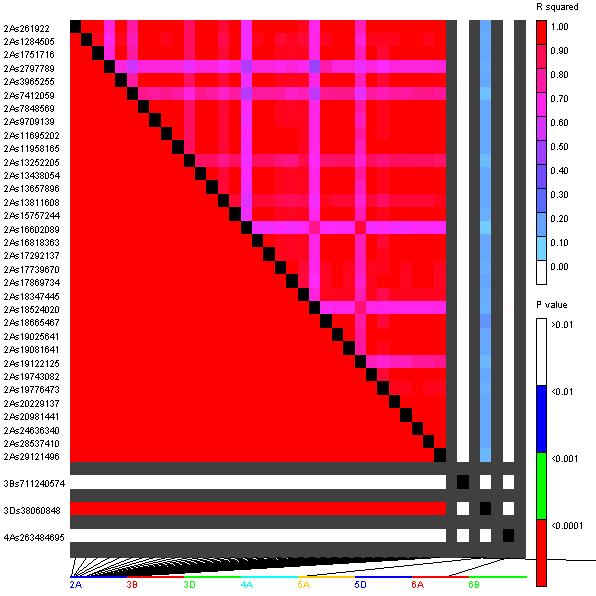

Supplement: Supplementary file 1 [file plants-10-02693-s001.zip › plants-1455551-supplementary/Supplementary Figure S2.jpg]
